# Apolipoprotein E ε4 Is Associated With the Development of Incident Dementia in Cerebral Autosomal Dominant Arteriopathy With Subcortical Infarcts and Leukoencephalopathy Patients With p.Arg544Cys Mutation

**DOI:** 10.3389/fnagi.2020.591879

**Published:** 2020-11-20

**Authors:** Jung Seok Lee, Keun Hyuk Ko, Jung-Hwan Oh, Joong-Goo Kim, Chul-Hoo Kang, Sook-Keun Song, Sa-Yoon Kang, Ji-Hoon Kang, Joon Hyuk Park, Myeong Ju Koh, Ho Kyu Lee, Jay Chol Choi

**Affiliations:** ^1^Department of Neurology, Jeju National University, Jeju, South Korea; ^2^Department of Neurology, Hankook Hospital, Jeju, South Korea; ^3^Department of Neurology, Jeju National University Hospital, Jeju, South Korea; ^4^Department of Psychiatry, Jeju National University, Jeju, South Korea; ^5^Department of Radiology, Jeju National University, Jeju, South Korea; ^6^Institute of Medical Science, Jeju National University, Jeju, South Korea

**Keywords:** CADASIL, dementia, MRI, APOE genotyping, stroke

## Abstract

**Background and Purpose:**

To identify clinical, laboratory, and magnetic resonance imaging (MRI) features in predicting incident stroke and dementia in Korean patients with cerebral autosomal dominant arteriopathy with subcortical infarcts and leukoencephalopathy (CADASIL).

**Materials and Methods:**

We enrolled 87 Korean CADASIL patients who had undergone baseline clinical, laboratory, and MRI examinations between March 2012 and February 2015. The primary outcome of this study is the occurrence of stroke and dementia during the study period. The occurrence of incident stroke was confirmed by neuroimaging study, and dementia was defined by the diagnostic and statistical manual of mental disorders, fourth edition, criteria.

**Results:**

Of the 87 patients, 57.5% were men, and the mean age was 63 ± 13 years (range 34–90 years), and 82 patients (94.3%) had p.Arg544Cys mutation. During an average follow-up of 67 months (interquartile range: 53–69 months), incident stroke occurred in 14 of 87 patients (16.1%) and incident dementia in 7 of 70 non-demented patients (10.0%). In adjusted analysis, increased systolic blood pressure was associated with increased risk of incident stroke [for every 10-mmHg increase; hazard ratio, 1.44 (1.02–2.03)]. Apolipoprotein E ε4 genotype was associated with an increased risk of incident dementia [hazard ratio, 10.70 (1.27–89.88)].

**Conclusion:**

In this study, apolipoprotein E ε4 genotype was associated with the development of incident dementia, and higher blood pressure was associated with increased risk of incident stroke in CADASIL patients with predominant p.Arg544Cys mutation.

## Introduction

Cerebral autosomal-dominant arteriopathy with subcortical infarcts and leukoencephalopathy (CADASIL) is the most common inherited cerebral small vessel disease caused by mutations in the *NOTCH3* gene. The main clinical manifestations are recurrent stroke, vascular cognitive decline, chronic headache, and mood disturbances. Ischemic stroke is the most frequent clinical manifestation of CADASIL and was found in 60–84% of Caucasian CADASIL patients (Chabriat et al., 1995; Desmond et al., 1999; Monet-Lepretre et al., 2009). The average incidence of new stroke was estimated to be 10.4 per 100 person-years in CADASIL (Peters et al., 2004).

Cognitive deficit is the second most common clinical feature in CADASIL, and dementia has been reported to be present in 16–38% of the patients with the mean age of onset in the mid-50s (Dichgans et al., 1998; Adib-Samii et al., 2010; Bianchi et al., 2015). CADASIL patients show an early frontal executive dysfunction similar to sporadic subcortical small vessel disease, and these cognitive deficits can be discovered by neuropsychological testing before the occurrence of ischemic stroke (Buffon et al., 2006; Charlton et al., 2006). The severity of cognitive deficit is closely associated with the number of lacunar infarctions and the degree of brain atrophy seen in brain magnetic resonance imaging (MRI) (Viswanathan et al., 2007; Liem et al., 2009; Chabriat et al., 2016). Several previous case reports suggested the coexistence of amyloid pathology in patients with CADASIL, although CADASIL is known as a typical model of pure subcortical vascular dementia (Thijs et al., 2003; Paquet et al., 2010; Yoon et al., 2015). Therefore, we hypothesized that amyloid pathology could affect the development of dementia in patients with CADASIL.

Apolipoprotein E (APOE) is the most abundant apolipoprotein in the brain (Roher et al., 2009) and has been suggested as a chaperone of beta-amyloid deposition or clearance (Aleshkov et al., 1997). Several studies have demonstrated that the APOE ε4 allele is an independent risk factor for Alzheimer’s dementia (Strittmatter et al., 1993). Besides, the associations with the APOE have been observed in several other central nervous system diseases, such as dementia with Lewy bodies, recovery of stroke, and risk of cognitive impairment after chemotherapy (Flowers and Rebeck, 2020). However, the relationship between the APOE ε4 allele and vascular dementia is still controversial (Zekry et al., 2010). In CADASIL, a large cross-sectional study reported from the United Kingdom did not report a significant association between APOE ε4 allele and dementia (Singhal et al., 2004).

The clinical course of CADASIL varies widely, and several previous research suggested distinct genotypic and phenotypic profiles in Asian CADASIL patients (Opherk et al., 2004; Choi et al., 2006, 2013; Lee et al., 2009; Liao et al., 2015). Several cardiovascular risk factors and brain MRI features can help predict the clinical course of CADASIL (Liem et al., 2009; Adib-Samii et al., 2010; Chabriat et al., 2016; Jouvent et al., 2016; Ling et al., 2019). However, predictors of recurrent stroke or dementia have not been investigated in-depth among Asian CADASIL patients whose clinical course might be different from that of Caucasian patients. This study aimed to identify clinical, laboratory, and brain MRI features in predicting incident stroke and dementia in Korean patients with CADASIL.

## Materials and Methods

### Study Cohort

The cohort consisted of 94 patients who had been genetically confirmed as CADASIL and undergone baseline clinical, laboratory, and MRI examinations between March 2012 and February 2015 (Lee et al., 2017). For this study, we included CADASIL patients who had been followed up regularly for at least 36 months. For the patients who were not followed for 36 months, we contacted them by phone interview at the end of December 2018 for the occurrence of clinical events. This study was approved by the Institutional Review Board of Jeju National University Hospital, and all subjects gave written informed consent in accordance with the Declaration of Helsinki at the time of baseline examination. The details of the cohort have been published previously (Lee et al., 2017). In brief, we collected data on demographic and clinical characteristics, vascular risk factors, antithrombotic medication, and APOE genotyping. Clinical manifestations, including stroke (ischemic or hemorrhagic), cognitive dysfunction, psychiatric illness, recurrent headache, or seizure, were assessed at baseline and during follow-up. Blood pressure was measured using an automated sphygmomanometer (HBP-1300, Omron Healthcare, Kyoto, Japan) at the upper arm in the sitting position after the patient rested for at least 5 min. Activities of daily living were measured using Seoul-Instrumental Activities of Daily Living (Ku et al., 2004), and cognitive function was assessed using the Korean version of Mini-Mental State Examination and the Korean version of the Consortium to Establish a Registry for Alzheimer’s Disease Assessment Packet (Kang et al., 1997; Lee et al., 2002).

The primary outcome of this study is the occurrence of stroke and dementia during the study period. Stroke is defined as an acute neurologic deficit of vascular origin confirmed by appropriate neuroimaging study and was classified as an ischemic or hemorrhagic stroke. Dementia is defined according to the diagnostic and statistical manual of mental disorders, fourth edition, criteria. Incident strokes were recorded regardless of whether the patients had a stroke at the time of baseline examination. In the case of incident dementia, however, patients who had already been diagnosed with dementia in the baseline were excluded.

### Magnetic Resonance Imaging

We performed the MRI study acquired on a 3-T scanner (Achieva, Philips Healthcare, Best, Netherlands) using a 32-channel array head coil, as described in our previous study (Lee et al., 2017). The imaging analysis was performed independently by an experienced neuroradiologist (HL) and a stroke neurologist (KK) operating by consensus and without any knowledge of clinical information. Lacunar infarcts were defined as parenchymal defects not extending to the cortical gray matter with a signal intensity of cerebrospinal fluid in all sequences and more than 2 mm in diameter. Lesions located in the lower third of the corpus striatum of the basal ganglia were excluded (Bokura et al., 1998). Cerebral microbleeds (CMBs) were defined as small, rounded, or circular, well-defined hypodense lesions within brain parenchyma with clear margins ranging from 2 to 10 mm in size on T2^∗^-weighted images according to the microbleed anatomical rating scale (Gregoire et al., 2009). White-matter hyperintensities (WMHs) were defined as white-matter areas with increased signal intensities on fluid-attenuated inversion recovery images. All fluid-attenuated inversion recovery axial sections from the base of the cerebellum to the vertex were analyzed. WMH volume was calculated automatically, as previously described (Jeon et al., 2011; Kim et al., 2015). Estimated total intracranial volume (ICV) was determined automatically on the three-dimensional T1-weighted images by using FreeSurfer 6.0^1^ (Fischl, 2012). In addition to the volumetric measurement, we used the Scheltens scale as a visual rating scale for WMH severity (Scheltens et al., 1993). All the Scheltens scale was rated by one of the contributing authors (C-HK). The brain parenchymal fraction (BPF), image parameter of brain atrophy, was defined as brain parenchymal volume to ICV. WMH volume was normalized for total brain volume by dividing the individual WMH volume by ICV [normalized WMH volume (nWMH)].

### Apolipoprotein E Genotyping

Genomic DNA was extracted from ethylenediaminetetraacetic acid-anticoagulated whole blood using QuickGene (Kurabo Industries Ltd., Osaka, Japan) according to the manufacturer’s instructions. For the multiplex polymerase chain reaction based on the dual priming oligonucleotide technology, the Seeplex Apo E ACE genotyping assay (Seegene, Seoul, South Korea), 17 μl of the master mix was remixed with 3 μl of genomic DNA. After a preheating step at 94°C for 15 min, 35 reaction cycles (denaturation at 94°C for 30 s, annealing at 65°C for 30 s, and extension at 72°C for 60 s) were performed and post-step at 72°C for 10 min using MyCycler Thermal Cycler System (Bio-Rad Laboratories, Irvine, CA, United States).

For the one-stop Real-Q ApoE genotyping assay, we mixed 21 ml of the master mix (including 17.5 ml of the reaction mixture, 3 ml of a primer mixture for codon 112 and 158, and 0.5 ml of sterile water) with 4 ml of genomic DNA. After the preheating step at 95°C for 10 min, 40 reaction cycles (denaturation at 95°C for 20 s, annealing at 60°C for 30 s, and extension at 72°C for 30 s) were performed on the 7500 Real-Time PCR System (Applied Biosystems, Foster City, CA, United States), and the results were analyzed by the standard curve method.

### Statistical Analysis

To investigate the factors associated with incident stroke or dementia during the study period, we used the time to event analysis. All covariates were first tested by unadjusted analyses using the log-rank test or Cox proportional hazard model according to the type of variables and subsequent adjusted analyses. Brain MRI variables entered the model after they were dichotomized by the median values. In multivariable analyses, age and sex were entered into the models in all analyses. Other covariates were included in the adjusted models if they had a *P*-value <0.10 in univariate analysis. For incident dementia, the analysis was limited to the patients who did not have dementia at baseline examination. Cox proportional-hazards model was used to adjust for covariates in the time to event analysis for incident dementia. Because the incident stroke recurred in some patients, we used an extension of the traditional Cox proportional-hazards model to evaluate the impact of multiple covariates for recurrent events (Prentice et al., 1981). For both multivariable analyses, three models were tested. Model 1 included demographic characteristics, clinical predictors, and APOE genotyping; model 2 included brain imaging markers; and model 3 included all covariates in model 1 and model 2 (Chabriat et al., 2016). As a sensitivity analysis, we repeated the survival analyses with the 82 patients having p.Arg544Cys mutation. All statistical analyses were performed using the Stata data analysis software (Version 15.1, StataCorp., College Station, TX, United States). In all analyses, a *p*-value <0.05 was considered statistically significant.

## Results

Of 94 patients who had been genetically confirmed as CADASIL and been evaluated at baseline, we excluded five patients in whom quantitative image analysis was not possible due to various reasons such as the presence of huge brain lesions or motion artifacts. Of the remaining 89 patients, 72 patients were regularly assessed at the outpatient clinic for at least 36 months; 15 patients who were not assessed at least for 36 months at the outpatient clinic were contacted by phone interview, and two patients who could not be reached were excluded in this study. As a result, we enrolled 87 patients from 72 unrelated families for the current analysis.

**FIGURE 1:**
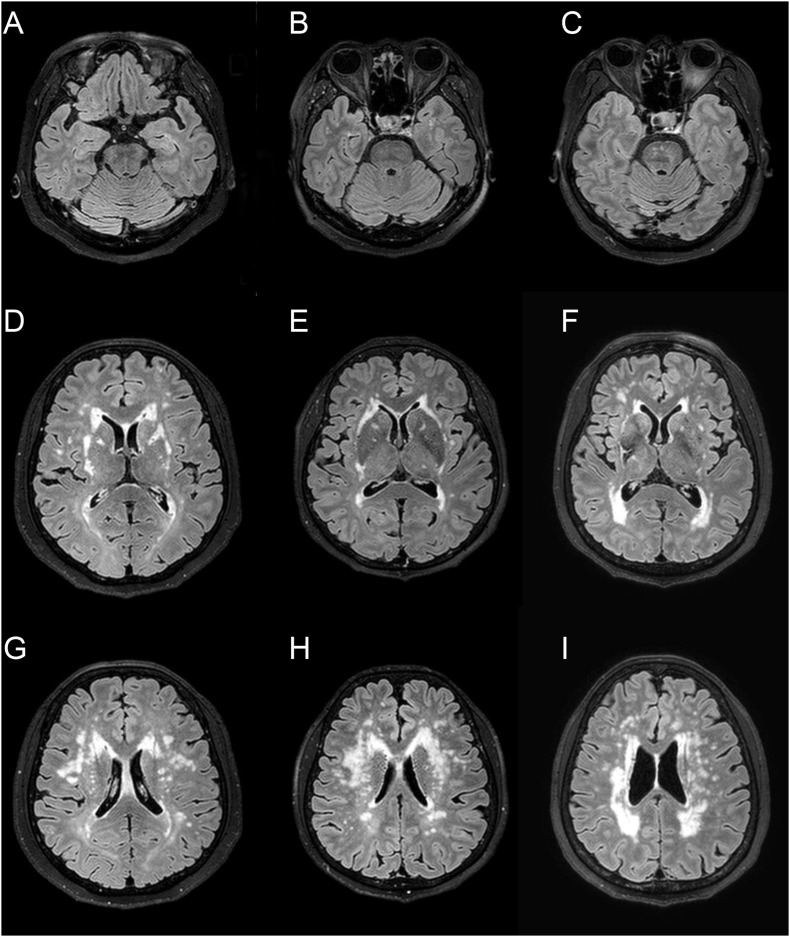
Representative fluid-attenuated inversion recovery MRI of three CADASIL patients with different APOE genotype. Patient 1 **(A,D,G)** is a 66-year-old female with APOE ε3/ε3 [normalized volume of white-matter hyperintensities (nWMH) = 0.0048]. Patient 2 **(B,E,H)** is a 67-year-old female with APOE ε2/ε3 (nWMH = 0.0051). Patient 3 **(C,F,I)** is a 66-year-old female with APOE ε3/ε4 (nWMH = 0.0049). All patients had p.Arg544Cys mutation.

The characteristics of the patients are summarized in Table 1. There were 50 men (57.5%), and the mean age was 63 ± 13 years (range 34–90 years). The median follow-up month was 67 months (interquartile range: 53–69 months). The APOE genotype frequencies were ε2/ε2 0 (0%), ε2/ε3 8 (9.2%), ε2/ε4 2 (2.3%), ε3/ε3 53 (60.9%), ε3/ε4 23 (26.4%), and ε4/ε4 1 (1.2%). Seventeen patients (19.5%) identified through family screening were asymptomatic. They had a mean age of 52.4 years (range 34–80 years), and 9 (52.9%) were male. The symptomatic patients (70 patients, 80.5%) had a mean age of 65.0 years (range 41–90 years), and 41 (58.6%) were male. Of the 87 patients, 43 patients (49.4%) had a history of ischemic stroke (34 patients) or a hemorrhagic stroke (15 patients), and 17 patients (19.5%) had been diagnosed with dementia at baseline. Ischemic stroke was the most frequent manifestation (*n* = 38, 43.7%), followed by cognitive impairment (*n* = 27, 31.0%), intracerebral hemorrhage (ICH) (*n* = 15, 17.2%), headache (*n* = 7, 8.0%), psychiatric symptom (*n* = 12, 13.8%), and seizure (*n* = 1, 0.9%). The APOE genotype was not associated with the normalized volume of WMH in unadjusted and adjusted analyses (Table 2 and Figure 1).

**TABLE 1:** Characteristics of the patients.

**Characteristics**	**(*n* = 87)**
**Demographic characteristics**	
Age, year (mean ± SD)	62.5 ± 12.7
Male sex, no. (%)	50 (57.5)
***NOTCH3* mutation, amino acid**	
R544C	82 (94.3)
R578C	2 (2.3)
R75P	2 (2.3)
C455R	1 (1.1)
**Apolipoprotein E** ε**4**	26 (29.9)
**Medical history, no. (%)**	
Hypertension	47 (54.0)
Diabetes mellitus	13 (14.9)
Atrial fibrillation	3 (3.4)
Hyperlipidemia	19 (21.8)
Current smoking	34 (39.1)
History of stroke	38 (43.7)
History of coronary artery disease	4 (4.6)
**Medication history, no. (%)**	
Antiplatelet agent	57 (65.5)
Oral anticoagulants	2 (2.3)
**Clinical manifestations**	
Ischemic stroke	38 (43.7)
Cognitive impairment	27 (31.0)
Intracerebral hemorrhage	15 (17.2)
Psychiatric symptoms	12 (13.8)
Recurrent headache	7 (8.0)
**Laboratory values, mean ± SD**	
Systolic blood pressure, mmHg	123.9 ± 12.2
Diastolic blood pressure, mmHg	75.0 ± 10.7
Total cholesterol, mg/dl	174.3 ± 33.7
LDL cholesterol, mg/dl	103.0 ± 29.0
HbA1c, %	6.0 ± 0.7
Homocysteine, μmol/L	11.6 ± 6.0
**Disability and cognitive scores**	
S-IADL score, median (IQR)	1 (0–5)
K-MMSE score, median (IQR)	25 (20–27)
**MRI markers, mean ± SD, IQR, median**	
Brain parenchymal fraction	0.681 ± 0.123, 0.624–0.772, 0.681
Normalized volume of WMH	0.007 ± 0.008, 0.002–0.009, 0.005
No. of CMB	10.5 ± 17.8, 0–13, 4
No. of LI	4.7 ± 4.8, 1–8, 3

*S-IADL, Seoul-instrumental activities of daily living; K-MMSE, Korean version of the mental status examination; IQR, interquartile range; WMH, white matter hyperintensities; CMB, cerebral microbleeds; LI, lacunar infarction.*

**TABLE 2:** Effects of APOE genotype on normalized volume of WMH.

	**ε3/ε3, *n* = 53**	**ε2 carriers, *n* = 8**	**ε4 carriers, *n* = 24**	***P*-value**
Age, median (IQR) (years)	62 (50–72)	62 (61–65)	70 (56–75)	0.198
Sex, male, *n* (%)	31 (58.5)	5 (62.5)	12 (50.0)	0.735
Hypertension, *n* (%)	28 (52.8)	6 (75.0)	12 (50.0)	0.448
Diabetes mellitus, *n* (%)	8 (15.1)	3 (37.5)	2 (8.3)	0.139
Hyperlipidemia, *n* (%)	10 (18.9)	2 (25.0)	7 (29.2)	0.593
Smoking, *n* (%)	20 (37.7)	5 (62.5)	7 (29.2)	0.242
History of stroke, *n* (%)	25 (47.2)	5 (62.5)	12 (50.0)	0.720
Normalized volume of WMH, median (IQR)	0.005 (0.002–0.008)	0.005 (0.002–0.008)	0.006 (0.003–0.008)	0.648

*IQR, interquartile range; WMH, white matter hyperintensities.*

**FIGURE 2:**
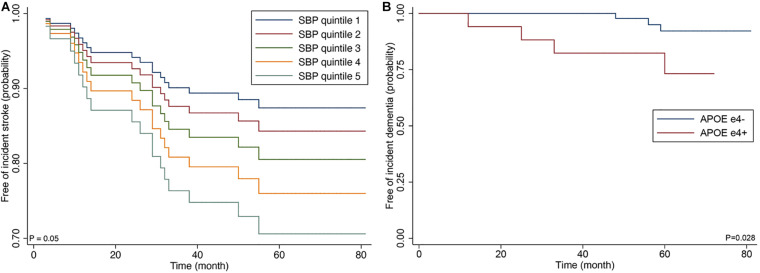
Cox proportional hazards regression curve by the quintiles of systolic blood **(A)** and Kaplan-Meier curve by APOE ε4 genotype **(B)**.

Incident stroke occurred in 14 of 87 patients (16.1%); 13 patients (14.9%) experienced new ischemic strokes, and two patients developed hemorrhagic strokes. Of 13 patients who developed new ischemic stroke, four patients (30.8%) experienced recurrent ischemic strokes. In the Cox proportional-hazards model, systolic blood pressure (SBP) was at baseline (model 1); higher nWMH and CMB number ≥5 (model 2); SBP and CMB number ≥5 (model 3) was significantly associated with increased risk of incident stroke (Table 3 and Figure 2). The sum of Scheltens scales was not associated with the risk of incident stroke in the unadjusted analysis [hazard ratio (HR) 1.33, 95% confidence interval (CI) 0.46–3.82, *P* = 0.596] in contrast to the volumetric assessment.

**TABLE 3:** Predictors of incident stroke and dementia.

	**Model 1**			**Model 2**			**Model 3 (1 + 2)**		
**Events**	**Predictors**	**HR (95% CI)**	***P*-value**	**Predictors**	**HR (95% CI)**	***P*-value**	**Predictors**	**HR (95% CI)**	***P*-value**
Stroke	SBP (per 10 mmHg increase)	1.59 (1.12–2.29)	0.01	nWMH volume (≥0.005)	6.69 (1.14–39.20	0.035	SBP (per 10 mmHg increase)	1.44 (1.02–2.04)	0.038
				No of CMB (≥5)	12.80 (1.81–90.84)	0.011	No. of CMB (≥5)	10.77 (1.33–87.02)	0.026
Dementia	Age	1.10 (1.01–1.20)	0.032	–			APOE ε4	10.70 (1.27–89.88)	0.029
	APOE ε4	8.53 (1.43–50.70)	0.018	–					

*HR, hazard ratio; SBP, systolic blood pressure; nWMH, normalized white matter hyperintensities; CMB, cerebral microbleeds; APOE, apolipoprotein E.*

*Model 1 included demographic characteristics, clinical predictors, and APOE genotyping; model 2 included brain imaging markers; and model 3 included all covariates in model 1 and model 2.*

Of the 87 patients, the presence of APOE ε4 genotype was associated with dementia at baseline examination (odds ratio 3.51; 95% CI 1.01–12.1; *p* = 0.021). Incident dementia developed in 7 patients (10.0%) among 70 CADASIL patients who did not have dementia at baseline examination. In the Cox proportional-hazards model, advanced age and presence of APOE ε4 genotype were associated with increased risk of incident dementia among clinical predictors (model 1) (APOE ε4; HR 8.53; 95% CI 1.43–50.70; *p* = 0.018), and APOE ε4 was correlated with increased risk of dementia after the adjustment for MRI predictors (model 3) (HR 10.7; 95% CI 1.27–89.88; *p* = 0.029).

**FIGURE 3:**
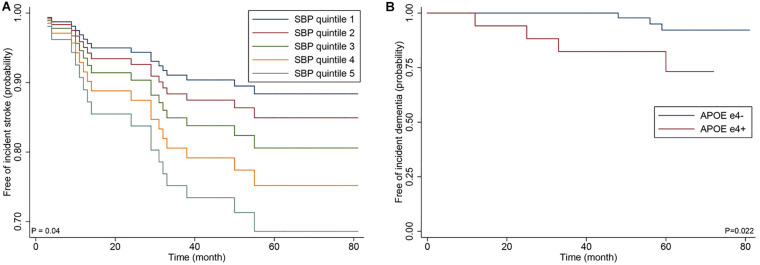
Cox proportional hazards regression curve by the quintiles of systolic blood **(A)** and Kaplan-Meier curve by APOE ε4 genotype **(B)** in the 82 patients with p.Arg544Cys mutation.

In the sensitivity analyses of the 82 patients with p.Arg544Cys mutation, SBP, and APOE ε4 genotype remained significant for increased risk of incident stroke (HR 1.49; 95% CI 1.08–2.06; *p* = 0.016) and dementia (HR 10.97; 95% CI 1.41–85.61; *p* = 0.022) in adjusted analyses (Figure 3).

The sum of the Scheltens scale was not associated with the increased risk of incident stroke or dementia. During more than 5 years of average follow-up, three patients (3.45%) had died, and the causes of death were lung cancer, pneumonia, and severe stroke.

## Discussion

The most important finding of the current study is that APOE ε4 allele was an independent risk factor for developing incident dementia in CADASIL patients. Recent studies have shown that APOE ε4 is not only a major risk factor for Alzheimer’s disease but also an independent risk factor for progression to severe cerebral amyloid angiopathy and cerebral amyloid angiopathy-related cerebral hemorrhage (O’Donnell et al., 2000; Bu, 2009). A large cross-sectional study reported from the United Kingdom did not find a correlation between APOE ε4 allele and dementia in patients with CADASIL (Singhal et al., 2004). Therefore, the effects of APOE ε4 on ongoing cognitive deterioration or development of dementia have not yet been reported in patients with CADASIL. For MRI markers of cerebral small-vessel disease in CADASIL patients, APOE ε4 did not show an independent association with CMB (Singhal et al., 2004; van den Boom et al., 2006; Lee et al., 2017). Only one study showed that CADASIL patients with APOE ε2 had more severe WMHs than those with APOE ε3ε3 (Gesierich et al., 2016). Using amyloid positron emission tomography, the coexistence of Alzheimer’s disease pathology had already been reported in two patients among 15 patients with *NOTCH3* variant (Yoon et al., 2015). Therefore, to the best of our knowledge, this is the first study to demonstrate that APOE ε4 allele was an independent risk factor for incident dementia in CADASIL.

Interestingly, the proportion of the APOE ε4 genotype in this study is significantly higher than the proportion among the Korean control subjects reported in 2003 (29.9 vs. 17.6%) (Choi et al., 2003). From a global perspective, APO E ε4 frequencies varied widely from 0 to 49% depending on the population studied, and there is no clear explanation for such a wide variation (Eisenberg et al., 2010). All the patients enrolled in this study lived in Jeju island, and the presence of the predominant *NOTCH3* mutation suggested a founder effect. Therefore, a genetic drift associated with an isolated island population might explain a higher frequency of APOE ε4 allele in this study compared with the control subjects of the Korean peninsula. This finding needs to be reexamined among the general population of Jeju island in the future.

Because the mean age of our CADASIL patients is much higher than the Caucasian CADASIL patients, our CADASIL patients are more likely to have concomitant Alzheimer’s disease pathology. The increased mean age in our CADASIL cohort could be explained by a recent study on the effect of *NOTCH3* pathogenic variant on CADASIL disease variability. The study found that CADASIL patients with an epidermal growth factor like-repeat (EGFr) domain 7–34 pathogenic variant had 12-year-later age at onset of stroke and longer survival time than patients with an EGFr domain 1–6 variant (Rutten et al., 2019). The majority of Caucasian patients with CADASIL had an EGFr domain 1–6 pathogenic variant, whereas most of our patients had an EGFr domain 13 variant that corresponded to exon 11 (Rutten et al., 2016). Another recent systemic review suggested a significant correlation between the pathogenicity of *NOTCH3* mutations and phenotypic severity in CADASIL (Xiromerisiou et al., 2020). Therefore, the p.Arg544Cys mutation appears to cause a less severe CADASIL phenotype, and low pathogenicity of the mutation may allow APOE ε4 genotype to serve as a disease modifier among elderly CADASIL patients. Our findings suggest that APOE ε4 can be used as a marker for the development of dementia in elderly CADASIL patients with less pathogenic *NOTCH3* variants. Prospective cohort studies using amyloid positron emission tomography are needed to determine whether the cause of dementia is purely CADASIL or a combination with Alzheimer’s disease pathology in our patients with CADASIL.

Our study also demonstrated that BPF in patients at baseline was not associated with incident dementia at follow-up. This contrasts with the findings of a recent study showing that BPF independently predicted incident dementia among the Paris–Munich CADASIL cohort (Chabriat et al., 2016). However, an earlier Dutch CADASIL cohort study could not find a significant association between brain atrophy and cognitive decline (Liem et al., 2009). Lacunar infarction is a well-known predictor of cognitive decline in CADASIL (Liem et al., 2007), and brain atrophy seen in patients with CADASIL is related to age and volume of lacunar lesions (Jouvent et al., 2007). Compared with the Paris–Munich cohort, our CADASIL patients had a higher mean age (63 vs. 51 years) and greater brain atrophy (mean BPF 0.681 vs. 0.853) despite the same median number of lacunes. We assumed that the greater brain atrophy in our patients is probably due mainly to an advanced age because the number of lacunes was similar to that of the Paris–Munich cohort. Therefore, baseline BPF, unlike the Paris–Munich cohort, might not well predict cognitive decline in our cohort.

Another important finding of the current analysis is that higher SBP was strongly associated with increased risk of incident stroke. Hypertension is the most important risk factor for stroke in general (O’Donnell et al., 2010). However, the effects of blood pressure or history of hypertension on the clinical course of CADASIL have not been investigated extensively so far. For brain MRI markers of small-vessel disease, previous research had found significant associations between SBP and cerebral atrophy (Peters et al., 2006), CMB (Viswanathan et al., 2006), and WMH (Holtmannspotter et al., 2005) in patients with CADASIL. For incident stroke, hypertensive CADASIL patients showed more than the twofold increased risk of incident stroke in a large prospective cohort study from the United Kingdom, suggesting the importance of controlling cardiovascular risk factors such as blood pressure in modulating the clinical course of CADASIL (Adib-Samii et al., 2010). In line with the United Kingdom study, SBP at baseline was associated with a significantly increased risk of incident stroke in this study. Future studies should investigate the effects of long-term variations of blood pressure and the effects of controlling blood pressure on the risk of stroke in patients with CADASIL.

We further found that an increased number of CMB could predict incident stroke, including ischemic stroke in CADASIL patients. These results are in line with several previous cohort studies that suggested that CMBs are associated with subsequent risks of ischemic stroke and ICH in patients with a transient ischemic attack or cerebral infarction in general (Charidimou et al., 2013; Wilson et al., 2019). However, few cohort studies were conducted on incident stroke in CADASIL patients with CMBs at baseline. The Paris–Munich cohort has shown that the presence of CMBs was associated with subsequent stroke in the analysis model, which included only MRI parameters (Chabriat et al., 2016). However, this relationship disappeared when the analysis model included clinical, epidemiological factors, and MRI parameters. Advanced age, high prevalence of hypertension (54 vs. 19%), and the greater median number of CMBs (4 vs. 0) may explain the different impacts of the CMBs on the incident stroke between the Paris–Munich cohort and the current cohort. In patients with ischemic stroke in general, CMBs are associated with a greater relative hazard for subsequent ICH than for ischemic stroke (Wilson et al., 2019). However, the presence of CMBs should not discourage the use of antiplatelet therapy in patients with CADASIL because our CADASIL patients also showed a greater absolute risk of ischemic stroke than that of ICH, just like the patients with ischemic stroke in general.

There are several limitations to our study. First, compared with the European CADASIL cohort, the sample size was relatively small in our study, leading to wide CIs of the results. Large Asian CADASIL collaboration studies are needed to overcome this limitation. Second, p.Arg544Cys mutation in exon 11 accounted for 94.3% of the mutation in our study, which may not be generalized to CADASIL patients with other types of mutation. Third, 17% of the participants did not have enough outpatient clinical follow-up, and therefore, they were contacted by phone interview. Additionally, our patients had variable follow-up duration compared with earlier research (Chabriat et al., 2016). Accurate evaluation of cognitive function and mild incident stroke can be difficult with a phone interview. Finally, the patients did not have an independent evaluation of incident dementia and did not receive detailed cognitive tests at fixed intervals. The strength of our study is its longitudinal design, use of standardized brain imaging protocol, and the first Asian CADASIL study that investigated predictors of incident stroke and dementia.

## Conclusion

In conclusion, APOE ε4 genotype was associated with the development of incident dementia, and higher blood pressure was associated with significantly increased risk of incident stroke in CADASIL patients with predominant p.Arg544Cys mutation.

## Data Availability Statement

Anonymized data used for this study are available to qualified researchers on request to the corresponding author.

## Ethics Statement

The studies involving human participants were reviewed and approved by the Jeju National University Hospital IRB. The patients/participants provided their written informed consent to participate in this study.

## Author Contributions

JL, KK, and JC: conception and design of the study. JL, KK, J-HO, J-GK, C-HK, S-KS, S-YK, J-HK, JP, and JC: acquisition of data. MK and HL: interpretation of data. JL and JC: drafting a significant portion of the manuscript. All authors contributed to the article and approved the submitted version.

## Conflict of Interest

JC is a site investigator of multicenter clinical trials or clinical studies sponsored by Boehringer Ingelheim, AstraZeneca Korea, Jeil Pharmaceutical Company Ltd., and ChongKeunDang Corp., and received lecture honoraria from BMS Korea, Samjin Pharmaceutical Company Ltd., Bayer Korea, ChongKeunDang Corp., and Shire Korea Ltd. The remaining authors declare that the research was conducted in the absence of any commercial or financial relationships that could be construed as a potential conflict of interest.

## Footnotes

1 http://surfer.nmr.mgh.harvard.edu
